# An eco-friendly bioanalytical RP-HPLC method coupled with fluorescence detection for simultaneous estimation of felodipine and metoprolol

**DOI:** 10.1186/s13065-025-01507-0

**Published:** 2025-05-23

**Authors:** Mariam Wasim Beniamin, Amira Mohamed Kessiba, Maha Abdelmonem Hegazy, Ahmed Emad El Gendy, Lubna Ahmed Kormod

**Affiliations:** 1 Department of Pharmaceutical Chemistry, Faculty of Pharmacy, Misr International University, KM 28, Cairo, Egypt; 2 Department of Analytical Chemistry, Faculty of Pharmacy, Cairo University, Kasr El-Aini Street, Cairo, 11562 Egypt

**Keywords:** Felodipine, Metoprolol, Bioanalytical validation, Green chemistry

## Abstract

An ecofriendly, sensitive, selective, precise, and accurate HPLC method with fluorescence detection (FD) has been developed and validated for simultaneous determination of two hypertensive drugs, felodipine and metoprolol in their pure samples, combined pharmaceutical dosage form and in spiked human plasma. Separation was carried out on Inertsil C_18_ column (150 mm × 4.6 ID; Particle size 5 µm) and a mobile phase composed of ethanol and 30mM potassium dihydrogen phosphate buffer, adjusted to pH 2.5 using ortho-phosphoric acid (40:60, v/v) was used. A constant flow rate of 1.0 mL/min at an ambient temperature was adopted. Method validation was performed according to the ICH Q2 R2 and bioanalytical method validation was done according to FDA guidelines for determination of both drugs simultaneously in biological matrices. The method demonstrated excellent linearity over the concentration range of 0.01–1.00 µg/mL and 0.003–1.00 µg/mL that were selected with accordance to the C_max_ of both drugs with a correlation coefficient (r^2^) of 0.9998 and 0.9999 for felodipine and metoprolol, respectively in human plasma. The intra-day and inter-day precision were ≤ 2% in their pure forms and in spiked human plasma, and the accuracy was within ± 2% of the nominal concentration for both drugs in their pure forms and within ± 10% of the nominal concentration in human plasma. A statistical comparison was conducted between the proposed method and the reported method; it showed no significant difference between both methods regarding both accuracy and precision. The proposed method proved to be eco-friendly with three green assessment tools (AGREE calculator, MoGAPI, RGBfast study).

**Graphical Abstract:**

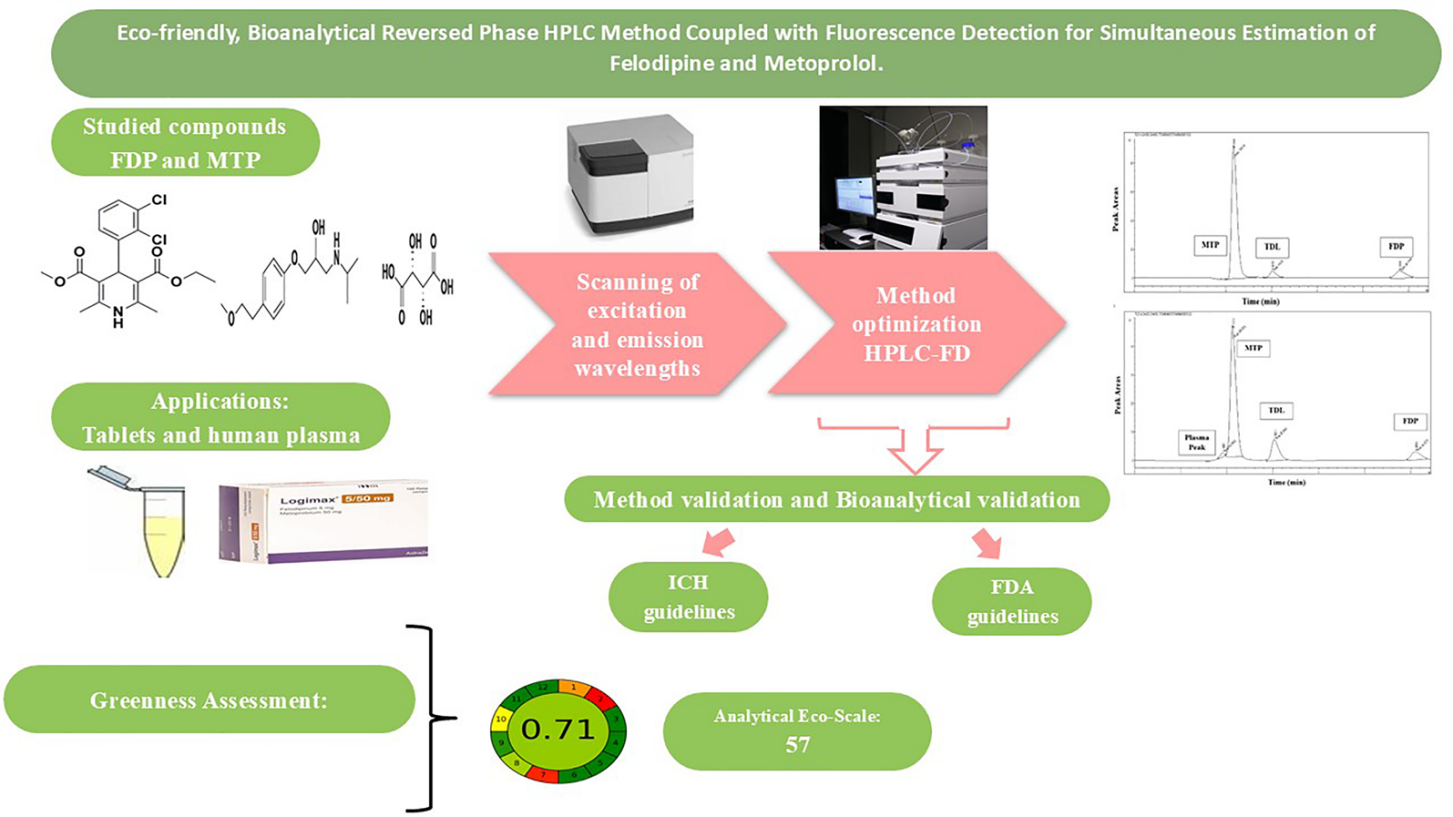

**Supplementary Information:**

The online version contains supplementary material available at 10.1186/s13065-025-01507-0.

## Introduction

Over the past decade, the World Health Organization (WHO) has been evaluating the prevalence of deaths from cardiovascular diseases globally. It was observable that cardiovascular diseases (CVDs) are the leading cause of death, mainly in low and middle-income countries. Mortality rate reached 17.9 million death per year worldwide resulting from CVDs; 85% were because of heart attack and stroke. As a result, the WHO is trying to raise the chances of survival through programs that permit patients to access technology and essential medications in various countries [1]. Essential medications that the cardiologists are concerned about encounter classes such as beta-blockers, statins, and blood thinners. Cardiovascular diseases include angina pectoris, myocardial infarction, coronary artery disease, and heart attack [2]. Angina pectoris and myocardial infarction are ischemic heart diseases characterized by insufficient blood flow pumped to the heart due to narrowed heart arteries, resulting in diminished oxygen and blood levels received by the heart muscle. The narrowing of blood vessels can be due to hypertension and/or hyperlipidemia [3].

Cardiovascular disease therapy depends on drug combinations from diverse classes. The American Heart Association guidelines claim that treating patients rely on numerous factors, including comorbidities that affect the overall disease state [4].

It is common in various diseases that the treatment requires to administer two or more drugs in the same pharmaceutical dosage form concomitantly which enhance patient compliance and make it easier to stick to the treatment regimen. This shows a greater impact in the treatment of various diseases [5] resulting in increased efficacy with less cost in a shorter time and patients compliance [6]. Correspondingly, various analytical techniques and methods have been implemented for multicomponent analysis, and the development of different methods is still in progress to guarantee high standards of quality and to ensure safety and efficacy [7].

Felodipine (FDP) is a second-generation calcium channel blocker [8] which is used in the management of angina pectoris and hypertension as a vasodilator and antiarrhythmic drug [9]. While metoprolol (MTP) is a selective beta blocker that reduces blood pressure used in the treatment of hypertension and arrythmia [10].

**Fig. 1:**
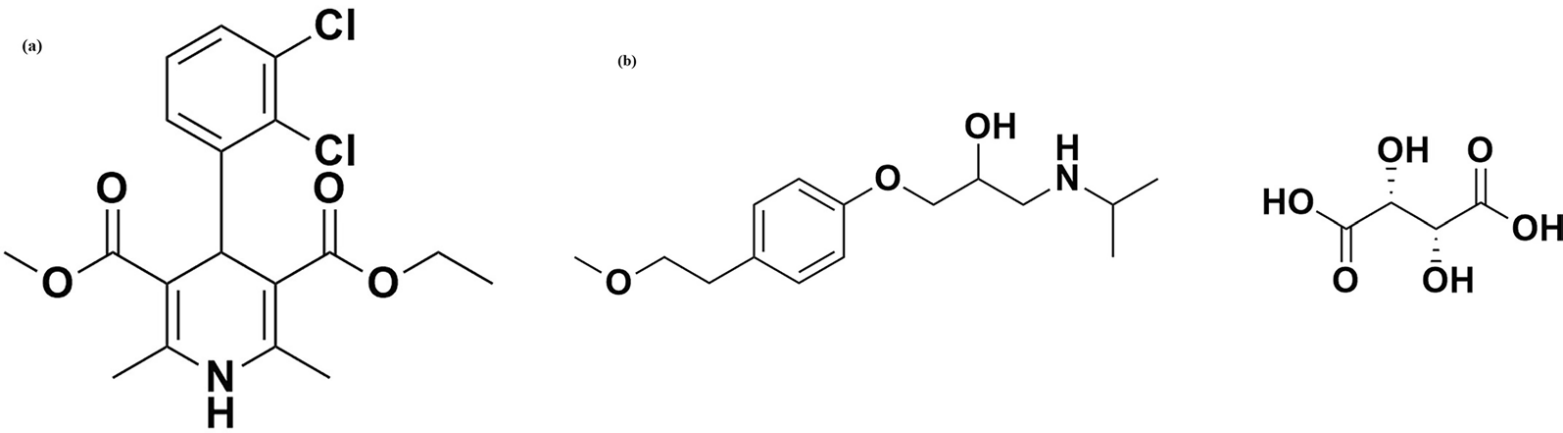
**a** Chemical structure of FDP. **b** Chemical structure of MTP

Chemically, FDP (C_18_H_19_Cl_2_NO_4_) is (methyl, ethyl) diester of 4-(2,3-dichlorophenyl)−2,6-dimethyl-1,4-dihydropyridine-3,5-dicarboxylic acid [11, 12] (Fig. 1a), while MTP tartarate (C_34_H_56_N_2_O_12_) is (2R,3R)−2,3-dihydroxybutanedioicacid;1-[4-(2-methoxyethyl)phenoxy]−3-(propan-2-ylamino)propan-2-ol [13, 14] (Fig. 1b).

Because of the increasing use of FDP and MTP in treatment of cardiovascular diseases and their strong existence in the pharmaceutical market globally [1], development of analytical methods became of increasing importance and several methods were reported.

The USP analytical method for estimating each drug separately is based on a chromatographic assay for FDP [15] and non-aqueous titration for MTP [16]. No official analytical methods are present for simultaneous determination of FDP and MTP. Several spectrophotometric methods have been reported for the simultaneous determination of FDP and MTP in bulk samples and pharmaceutical preparations. Rontogianni et al. [17] developed ratio spectra derivative and partial least square (PLS) methods, while Salem and Abdallah [18] proposed three simple spectrophotometric techniques: first derivative UV spectrophotometry with zero-crossing measurements, first derivative of ratio spectra, and PLS regression analysis. Additionally, Walash et al. [19] introduced a synchronous spectrofluorimetric method for the simultaneous determination of FDP and MTP.

In the field of thin layer chromatography, two high-performance thin layer chromatographic (HPTLC) methods have been reported for the determination of these drugs in dosage forms [20] and spiked human plasma [21].

Moreover, various high-performance liquid chromatography (HPLC) methods have been documented for analyzing FDP and MTP in bulk samples, pharmaceutical preparations [18, 21–24], and biological fluids [18, 21, 25]. HPLC is widely regarded as one of the most effective separation techniques due to its ability to provide excellent separation and quantification. When coupled with fluorescence detection (HPLC-FD), it can be applied either directly or indirectly, with most analysts preferring direct methods to avoid complex extraction and derivatization steps. HPLC-FD has emerged as a sensitive and cost-effective alternative to LC–MS/MS, offering highly accurate and sensitive quantification of drugs [26, 27].

Bioanalytical method validation is performed to guarantee a quantitative analytical method that exceed or meet the standards that have been provided and recommended by FDA Guidance [28] for known biomedical applications as bioequivalence, pharmacokinetic, and toxicokinetic study data. Assessing the stability during analysis while human plasma samples collection takes place is a crucial part in bioanalytical method validation [29, 30].

The present work introduces an eco-friendly bioanalytical simple, sensitive, accurate, selective, and economic HPLC method for simultaneous determination of FDP and MTP in pharmaceutical dosage form and in human plasma using Tadalafil (TDL) as an internal standard (IS).

## Experimental

### Instrumentation and software

An Agilent HPLC instrument (1200 series) Japan, bundled with Agilent Chemstation Chromatography software, equipped with an isocratic pump model G1310 A and connected with a fluorescence detector model G1321 A was used for the HPLC analysis. The injector was a manual Rheodyne injector (model G1328, USA).

A Shimadzu Spectrofluorometer (model: RF5301 PC, Japan) equipped with a 150-watt xenon lamp. Other instruments and devices include a pH meter (Jenway—3510—UK), a sonicator (Soniclean—120 T—Australia), and a membrane filter 0.45µm (Alltech—2024—USA).

### Chemicals and materials

Acetonitrile, ethyl alcohol, methyl alcohol, and ultrapure water were all HPLC grade and obtained from LiChrosolv^®^, Merck, Germany. with the following purities:

- Acetonitrile: ≥ 99.9%
- Ethyl Alcohol: ≥ 99.8%
- Methyl Alcohol: ≥ 99.8%
- Ultrapure Water: ≥ 18.2 MΩ·cm.

Potassium dihydrogen phosphate and ortho-phosphoric acid were kindly supplied from Adwic, Egypt with purities:

- Potassium dihydrogen phosphate: ≥ 99.0%
- Ortho-phosphoric acid: ≥ 85%

Felodipine was kindly supplied by Global Napi, Egypt, (batch number 002–171102) with certified purity of 99.81% as determined by the USP (United States Pharmacopeia) and BP (British Pharmacopoeia) methods. Metoprolol tartrate was kindly supplied by Inad Pharma, Egypt, (batch number 1021) with certified purity of 99.60% as by USP and BP methods. Tadalafil (TDL), the internal standard, was kindly supplied by El Andalus Medical Company (6^th^ of October city, Giza, Egypt) with certified purity 99.90% as determined by USP and BP methods. Logimax^®^ tablets manufactured by AstraZeneca, Egypt, (batch number 210361) were purchased from the local market and was labeled to contain 5 mg FDP and MTP succinate equivalent to 50 mg MTP tartrate per tablet. Human plasma was kindly supplied by the holding company for biological and vaccines VACSERA, Egypt

### Preparation of standard solutions and samples

#### Preparation of stock and working standard solutions

Stock standard solutions of 1.00 mg/mL FDP, MTP and TDL were prepared in minimum amount of methanol and volume was adjusted using ultra-pure water. Working standard solutions were prepared from the standard stock solutions by dilution with the mobile phase to obtain final concentrations 100.00 µg/mL, 10.00 µg/mL, and 1.00 µg/mL for FDP, MTP, and TDL, respectively. All solutions were kept at 5 ◦C.

#### Preparation of pharmaceutical formulation solutions

Ten tablets of Logimax^®^ were accurately weighed and finely powdered. The amounts of the powdered tablets equivalent to one tablet were accurately weighed, dissolved, then diluted to prepare final concentrations of 0.10 µg/mL of FDP and 1.00 µg/mL of MTP.

#### Preparation of quality control samples

Aliquots were transferred from working standard solutions of FDP and MTP, of 10.00 µg/mL concentration, into two 50-mL volumetric flasks, then diluted and adjusted to volume with ultrapure water to obtain quality control samples (Low, Mid, High) whose concentrations are 0.03, 0.50, and 0.90 µg/mL for FDP and 0.003, 0.009, 0.500 and 0.900 µg/mL for MTP together with constant concentration of TDL of 0.10 µg/mL.

#### Preparation of human plasma samples

Frozen human plasma was left to thaw at room temperature. In 5-mL stoppered centrifuge tubes, 980.0 µL of plasma were transferred. In each tube, 20.0 µL aliquots of FDP, MTP along with TDL working solutions were spiked. After spiking, the vortexing was done for 2.0 min at 1000 rpm. Protein precipitation technique was adopted for plasma treatment by adding 4.0 mL of acetonitrile to each of the stoppered centrifuge tubes, followed by centrifugation at 20 °C and 5000 rpm for 15.0 min. The supernatant was then filtered through 0.45 µm Millipore membrane filters.

### General procedure

#### Chromatographic conditions

For the analysis of both drugs, an Inertsil C_18_ column (150 mm × 4.6 ID; Particle size 5 µm) was used. Both drugs and the IS showed native fluorescence. The mobile phase of ethanol: 30 mM potassium dihydrogen phosphate buffer, adjusted to pH 2.5 using ortho-phosphoric acid, in the ratio (40:60, v/v) was used. A constant flow rate of 1.00 mL/min at an ambient temperature was used. Programing of excitation and emission wavelengths was adopted for each drug. MTP at and FDP were determined at excitation and emission wavelengths of 230.0, 300.0 nm and 367.0, 440.0 nm, respectively.

#### Construction of calibration curves

Calibration standards were prepared from FDP and MTP working standard solutions to give concentrations in the range of 0.01–1.00 µg/mL for FDP and 0.003–1.00 µg/mL for MTP, along with TDL fixed concentration of 0.10 µg/mL. Injections of 20.0 µL were done for each concentration in triplicates for the two drugs and the internal standard and chromatographic separation was obtained for each. The peak area ratios were calculated and plotted against their corresponding concentrations. Linear relationships were obtained for FDP and MTP, and the corresponding regression equations were computed. The analytical range to be validated was selected based on the plasma concentration of the studied analytes.

## Results and discussion

### Method development and optimization

#### Mobile phase preparation

Optimization of chromatographic conditions that include the chosen pH for analysis, organic solvent of mobile phase and wavelengths of detection were attained to elute peaks of high resolution and symmetry. The pKa values of the compounds of interest are 5.07 and 9.60 for FDP and MTP, respectively. Trials on different pH values were adopted, and the optimum pH was 2.5. FDP and MTP are weak bases with pKa values of 5.07 and 9.60, respectively. Below its pKa, FDP remains neutral and hydrophobic, strongly interacting with the stationary phase, leading to longer retention times. As pH nears 5.07, partial ionization reduces these interactions, shortening retention time. Above this pH, FDP becomes mostly ionized, further decreasing retention but potentially causing peak broadening [31]. Similarly, MTP remains protonated below pH 9.60, resulting in weak interactions and short retention times. As pH increases toward neutrality, MTP interacts more strongly with the column, increasing retention. However, high pH conditions may compromise column stability and peak shape, making them less ideal for analysis [32, 33]. Effect of different pH values upon several trials on the retention time presented in Fig. S1.

**Fig. 2:**
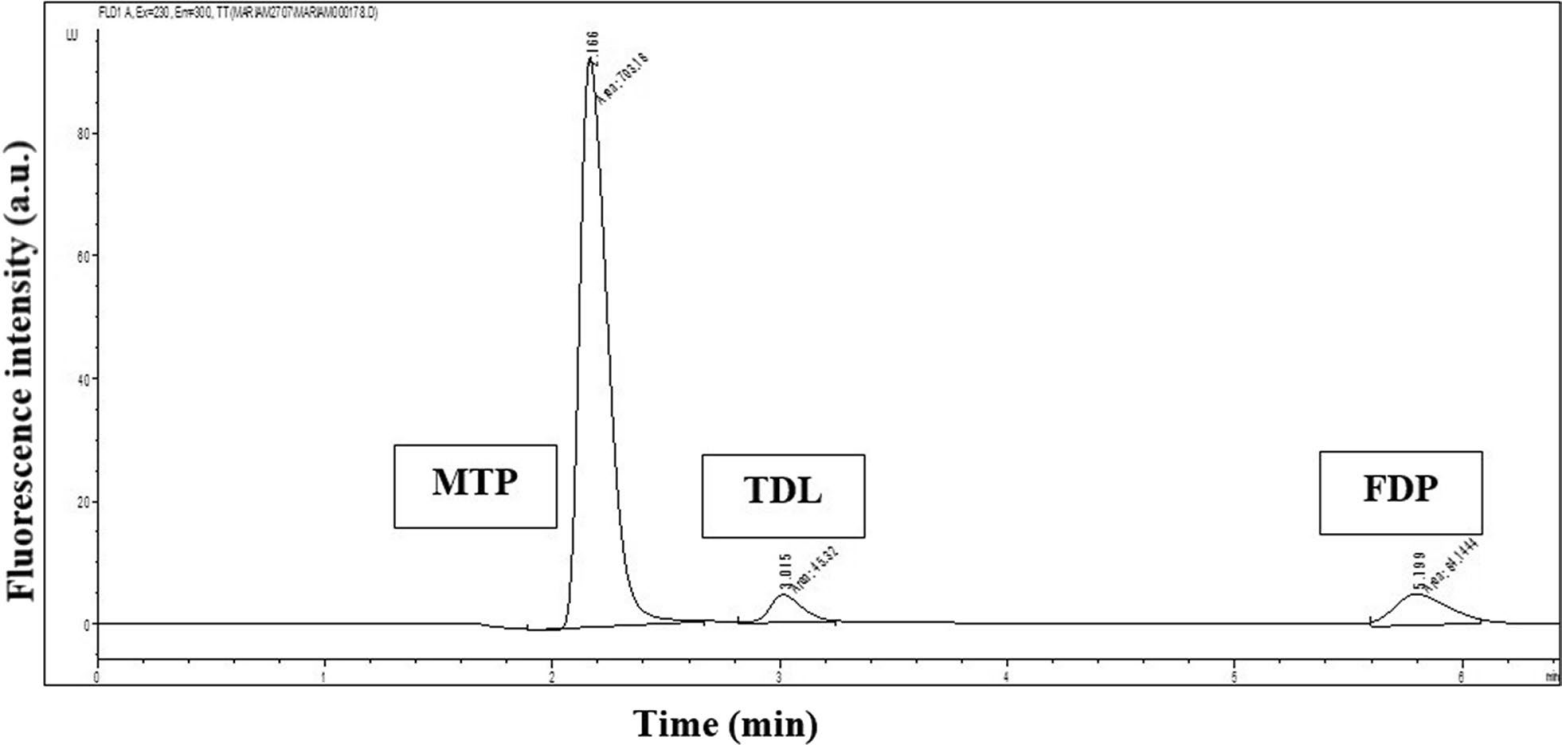
HPLC-FD chromatogram of metoprolol (MTP) (0.90 µg/mL), tadalafil (TDL) (0.10 µg/mL), and felodipine (FDP) (1.00 µg/mL) in their pure forms

Multiple of organic solvents were tested to achieve optimum separation in a short run time for both drugs. Ethanol was satisfactory as it is a green solvent yielding a satisfactory chromatogram. The ratio of the phosphate buffer to ethanol and the isocratic elution were optimized after different trials to obtain the best separation and resolution of the three compounds. Ethanol, as an organic modifier, reduces polarity and shortens retention times by weakening drug interactions with the stationary phase. The phosphate buffer maintains a stable pH of 2.5, ensuring optimal ionization for both drugs, improving peak shape, and enhancing separation. FDP, being more hydrophobic, interacts strongly with the column, leading to a longer retention time, while the more polar MTP elutes faster. This 40:60 ratio balances resolution, peak shape, and analysis time, making it ideal for effective separation. The various ethanol % that have been tested are demonstrated in Fig. S2. The resultant chromatogram of the analysis of the drugs in their pure form by the proposed HPLC-FD shown in Fig. 2, of both FDP and MTP along with TDL as an IS.

#### Plasma protein precipitation.

**Fig. 3:**
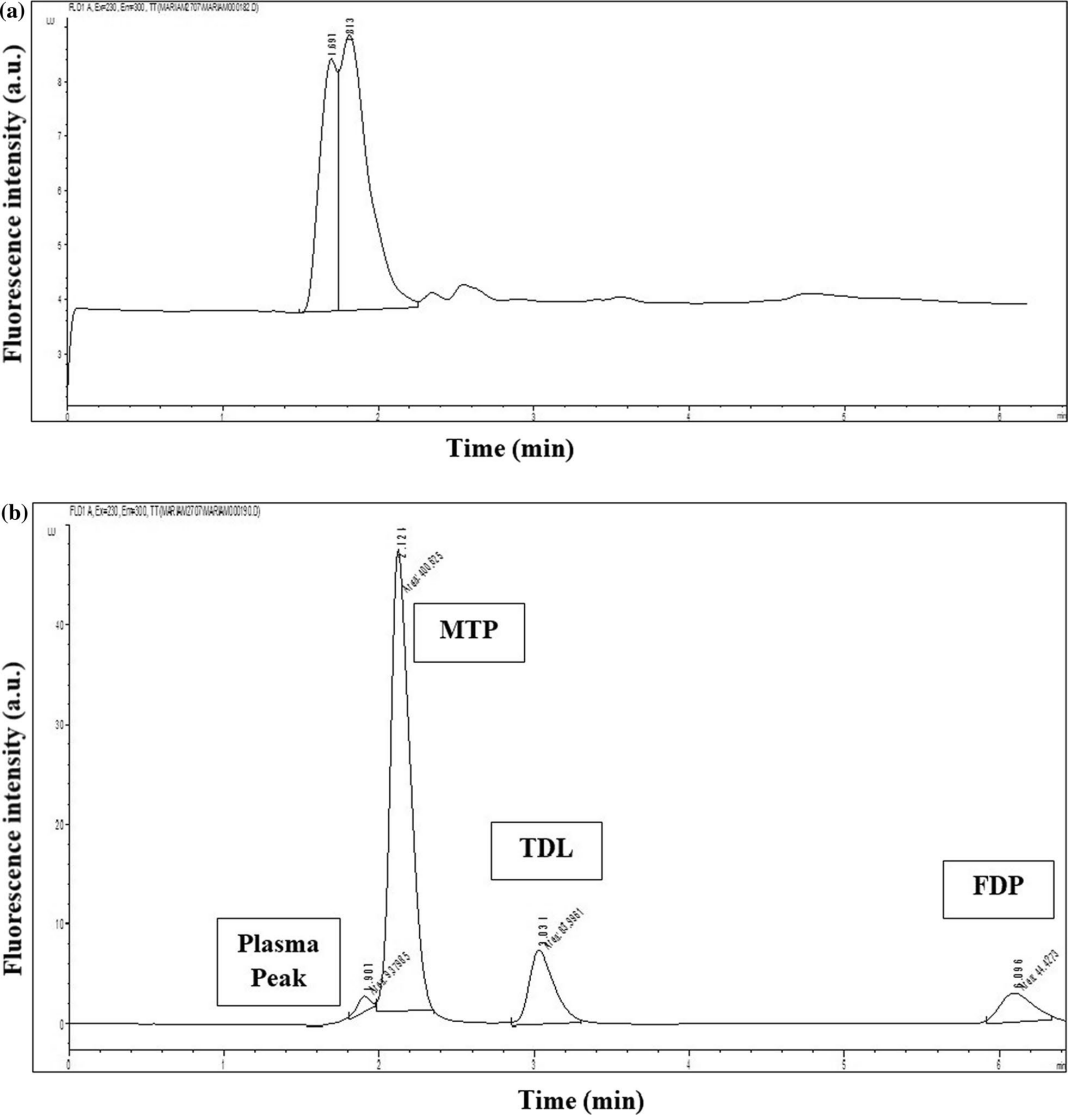
**a** HPLC-FD chromatogram of blank human plasma. **(b)** HPLC-FD of metoprolol, MTP (0.50 µg/mL), tadalafil, TDL (0.20 µg/mL), and felodipine, TDF (0.50 µg/mL) in spiked human plasma.

Human plasma treatment procedure adopted protein precipitation techniques. selection of the desired solvent that was used in protein precipitation rendered high reproducible recoveries. Selection of the solvent was based on many trials that resulted in choosing acetonitrile among methanol and ethanol that achieved the best recoveries. The employed chromatographic conditions and treatment in blank human plasma are shown in Fig. 3a, and the spiked human plasma is shown in the chromatogram in Fig. 3b. The two chromatograms were aligned on the same chromatogram to compare them directly. This makes it easy to see differences or similarities in retention times, peak shapes, and resolutions, as shown in Fig. S3.

### Method validation

#### Analytical validation

The validation of the proposed method was performed according to the ICH Q2 R2 [34, 35]. The regression and validation parameters of both drugs in their pure forms were summarized in Table 1.

**Table 1:** Regression and validation parameters for simultaneous determination of FDP and MTP in their pure forms

Parameters	FDP	MTP
**Linearity range** (µg/mL)	0.01–1.00	0.003–1.00
**Regression** • Slope • SE of slope • Intercept • SE of intercept • Correlation Coefficient (r)	1.859 0.0013 5.79 × 10^–5^ 0.00067 0.9999	19.554 0.0169 − 0.00092 0.0063 0.9999
**LOD** (µg/mL)	0.001	0.00096
**LOQ** (µg/mL)	0.004	0.0033
**Accuracy**: Mean ± SD	99.31 ± 0.803	99.38 ± 0.852
**Precision**: (%RSD) • Repeatability • Interday precision	0.880 1.338	0.755 0.977
**Specificity**: Mean ± SD	99.31 ± 0.200	99.14 ± 0.720

Bold represents the parameters and results that were further highlighted and focused on in the discussion of the results

##### Linearity

Aliquots of FDP and MTP working standard solutions were accurately transferred into 10-mL volumetric flasks, diluted, and completed to final volumes using ultrapure water to give concentrations in the range of 0.01–1.00 µg/mL for FDP and 0.003–1.000 µg/mL for MTP, along with TDL fixed concentration of 0.10 µg/mL. The analytical range to be validated was selected based on the peak plasma concentration (C_max_) of the studied analytes was found to be between 0.00096 and 0.0115 µg/mL for FDP [36]. While C_max_ of MTP was between 0.033 and 0.246 µg/mL [37]. Triplicate injections of 20.0 µL were done for each of the two drugs along with the internal standard. The relative peak areas were calculated and plotted against their corresponding concentrations illustrated in Fig. S4a, b. Linearity and parameters of the regression equation are listed in Table 1.

##### Accuracy

The previously mentioned procedure of linearity was applied to different concentrations within the linearity range of FDP and MTP along with TDL in their pure forms. The concentrations were calculated from the corresponding regression equations. The percentage recoveries, mean recoveries, and standard deviation were then calculated.

The quantitative recoveries of FDP and MTP in their pure forms achieved ranged from 98.33% to 100.00% and 98.33% to 100.00%, respectively. The mean recoveries and standard deviations are given in Table 1.

##### Repeatability and inter-day precision

The previously mentioned procedure of linearity was applied to freshly prepared triplicate injections of 0.03, 0.10, 0.90 µg/mL for both drugs. The percentage relative standard deviations were calculated. The assay was then repeated in three successive days for the same concentrations. The percentage relative standard deviation (% RSD) of FDP and MTP detected during the intraday and inter-day studies were all below 2% as described in Table 1.

##### Specificity

Aliquots from working standard solutions of FDP and MTP in different ratios, were used to prepare laboratory mixtures. The percentage recoveries, mean recoveries, and the standard deviation were calculated. The mean recoveries and standard deviations for lab mixtures are given in Table 1.

##### Limit of detection (LOD) and limit of quantification (LOQ)

The LOD was 0.001 μg/mL and 0.00096 μg/mL for FDP and MTP, respectively. While the LOQ was 0.004 μg/mL and 0.033 μg/mL for FDP and MTP, respectively as illustrated in Table 1.

##### System suitability

The tests for system suitability are an integral part of HPLC methods. These tests are applied to check that the chromatographic system is adequate for the intended analysis. Several parameters were studied according to the USP Pharmacopeia and national formulary [38] including retention time, Capacity factor, selectivity factor, resolution factor, asymmetry factor, number of theoretical plates, and height equivalent to theoretical plates. Satisfactory results are listed in Table 2.

**Table 2:** System suitability parameters for the proposed HPLC-FD method of FDP and MTP in their pure form

System suitability parameter	Metoprolol	Tadalafil (internal standard)		Felodipine	Theoretical values [35]
Retention time (min.)	2.166	3.015		5.799	> 1
Capacity factor (K′)	0.24	0.72		2.32	
Selectivity factor (α)	3.01			3.21	> 1
Resolution factor (R_s_)	5.42			13.49	> 2
Asymmetry factor	0.69	0.75		0.81	< 2
Number of theoretical plates (N)	3445	5303		8805	> 2000
Height equivalent to theoretical plates (HETP) (cm/Plate)	0.00435	0.00282		0.00170	The smaller the value, the higher the column efficiency

##### Robustness

The robustness of the developed method was carried out, several trials of different flow rates, Ethanol % and pH values were carried out. Retention times, calculation of resolution and number of theoretical plates showed no significant effect represented in Table 3 which proves the method’s capability to remain consistent in routine laboratory work.

**Table 3:** Robustness of the proposed HPLC-FD of FDP and MTP in their pure forms

Parameter	MTP			FDP		
	resolution factor (R_s_)	Number of theoretical plates (N)	RSD %	Resolution factor (R_s_)	Number of theoretical plates (N)	RSD %
Flow rate—0.1 mL/min (0.9 mL/min)	5.49	7146	0.853	18.33	9525	1.174
Flow rate + 0.1 mL/min (1.1 mL/min)	5.25	3451	0.992	11.72	10,766	1.651
Ethanol % + 2% (42%)	4.92	3671	0.759	9.28	8920	1.223
Ethanol %—2% (38%)	5.41	5890	0	10.39	6029	0.969
pH 2.6	5.39	3160	0.371	13.66	8130	0.157
pH 2.4	5.46	2998	0.229	14.09	7820	0.092

#### Bioanalytical validation

The bioanalytical validation of developed method in spiked human plasma was performed in accordance with the FDA guidelines [39].

##### Linearity

Both blank and spiked human plasma were injected under the developed chromatographic conditions. Calibration curves were constructed for FDP and MTP and regression equations were then computed. Calibration curves consisted of a blank sample (Blank plasma sample free from drugs as well as the I.S.), a zero caliber (Blank plasma sample injected and spiked with I.S) and six non-zero samples (0.01, 0.04, 0.05, 0.10, 0.60, 1.00 µg/mL for FDP and 0.003, 0.010, 0.040, 0.100, 0.600, 1.000 µg/mL for MTP) covering the expected concentration range including the LLOQ and ULOQ. Linearity and parameters of the regression equation of both drugs in human plasma are listed in Table 4.

**Table 4:** Regression of the proposed HPLC-FD method of FDP and MTP in spiked human plasma

Parameters	FDP	MTP
**Linearity range** (µg/mL)	0.01–1.00	0.003–1.00
**Regression** • Slope • SE of slope • Intercept • SE of intercept • Correlation Coefficient (r)	0.9596 0.0093 0.0516 0.0044 0.9998	9.3148 0.0541 0.0268 0.0258 0.9999

Bold represents the parameters and results that were further highlighted and focused on in the discussion of the results

##### Accuracy and precision for plasma samples

Accuracy and precision were obtained for LLOQ along with LQC, MQC, HQC (Low, Mid, and High) quality control samples. Five replicates of each concentration for each drug were analyzed on the same day to determine intraday precision and accuracy well as five replicate injections of same mentioned concentrations in three successive days to assess the inter-day precision and accuracy. Results are given in Table 5.

**Table 5:** Accuracy and Precision results of the proposed HPLC-FD method of FDP and MTP in spiked human plasma

Concentration (µg/mL)		FDP		Concentration (µg/mL)	MTP	
		Precision (RSD%)	Accuracy (recovery% ± SD)*			
					Precision (RSD%)	Accuracy (recovery% ± SD)*
Intra-day precision	0.01	1.024	98.77 ± 1.002	0.003	1.479	98.24 ± 1.727
	0.03	0.375	97.42 ± 0.293	0.009	0.778	97.46 ± 0.749
	0.50	0.604	99.66 ± 0.598	0.50	0.985	96.95 ± 0.880
	0.90	0.737	97.28 ± 0.620	0.90	0.376	98.06 ± 0.385
Inter-day precision	0.01	1.170	96.06 ± 1.115	0.003	1.753	98.77 ± 1.867
	0.03	0.539	97.58 ± 0.526	0.009	1.078	98.75 ± 1.129
	0.50	1.032	98.96 ± 1.049	0.50	1.636	98.67 ± 1.349
	0.90	0.928	98.33 ± 0.996	0.90	0.635	98.60 ± 0.630

* Mean of five replicate determination

##### Selectivity and specificity of spiked human plasma

During validation of the analytical method, it is crucial to check for the methods’ ability to differentiate between the analytes of interest and any interferences in the biological matrix. Comparison was done on a blank plasma Fig. 3a sample and a spiked plasma chromatogram Fig. 3b and was confirmed by absence of interferences at the retention times of FDP and MTP.

##### Recovery of plasma samples

The extraction recoveries were done to LLOQ along with LQC, MQC, HQC (Low, Mid, and High) quality control samples for each drug compared to extracts of blanks spiked with the analyte post extraction. (At Low, mid, high). Recovery studies were applied to the spiked human plasma through comparing peak area ratios of three freshly prepared extracted samples with other three unextracted standard solutions of the same concentrations. Recovery data was examined in triplicates using the concentrations LQC, MQC and HQC samples as recommended by FDA guidelines. Recovery results shown in Table 6.

**Table 6:** Recovery results of FDP and MTP in spiked human plasma by the proposed HPLC-FD method

Concentration (µg/mL)	FDP		Concentration (µg/mL)	MTP	
	Recovery* %	RSD%		Recovery* %	RSD%
0.01	96.72	0.198	0.003	97.29	0.843
0.03	97.94	0.728	0.009	91.05	0.738
0.50	98.87	0.952	0.50	97.83	1.328
0.90	95.98	0.689	0.90	98.34	0.594

* Mean of triplicate determination

##### Carryover effects

Injection of HQC sample for both drugs was performed, followed by injection of a sample with LLOQ. Assessment of peak area ratio of LLOQ that should be dependent on the analyte and independent of the preceding injection. Recovery % was calculated and found to be 92.00%.

##### Stability

Factors involving storage conditions, chemical properties of the drug and the matrix may affect drug stability in biological fluids. The stability of the drugs of interest in human spiked plasma was tested by the proposed HPLC-FD method as given in Table 7.

- **Short-term stability**

Three aliquots of each of LQC and HQC samples were thawed at room temperature and kept at room temperature for 4 to 6 hours and then analyzed.

- **Post Preparative stability**

Three replicates of the LQC and HQC samples were kept at room temperature for 24 hours, analyzed and compared to freshly prepared samples.

- **Freeze-thaw stability**

Three aliquots of LQC and HQC samples were stored at – 20 °C for 24 h and thawed at room temperature. The samples are refrozen under the same conditions. Analysis was performed per cycle and the process was repeated three times with at least 12 h freezing between cycles.

- **Long term stability**

Three aliquots of LQC and HQC samples were stored at – 20 °C for 30 days, analyzed and compared to samples of first day of long-term stability testing.

**Table 7:** Stability results of FDP and MTP in spiked human plasma by the proposed HPLC-FD method

Concentration (µg/mL)		FDP		Concentration (µg/mL)	MTP	
		RSD%	Recovery* %		RSD%	Recovery*%
Short-term	0.03	0.993	98.00	0.009	0.555	98.19
	0.90	1.793	97.69	0.90	1.181	98.52
Post preparative	0.03	0.352	98.65	0.009	0.602	98.63
	0.90	1.174	99.32	0.90	0.122	98.60
Freeze–thaw	0.03	0.353	98.30	0.009	0.690	98.57
	0.90	1.062	98.12	0.90	0.975	98.50
Long term	0.03	1.883	97.61	0.009	0.956	97.45
	0.90	0.888	99.59	0.90	1.199	98.61

*Mean of triplicate determination

### Application to pharmaceutical preparation:

The proposed method was applied to the pharmaceutical combination dosage form Logimax^®^ with percent recoveries 99.05 ± 0.949 and 99.12 ± 1.252 for FDP and MTP, simultaneously. Method’s accuracy was further assessed by standard addition technique. The % recovery, mean recovery and standard deviation shown in Table 8.

**Table 8:** Determination of FDP and MTP in Logimax^®^ tablets by the proposed HPLC-FD method and standard addition technique

Component	Found % ± SD^b^		Standard addition technique^a^			
		Taken (µg/mL)	Pure added (µg/mL)	Pure found (µg/mL)		Recovery^c^ %
FDP	99.05 ± 0.949	0.10	0.08	0.081		101.25
		0.10	0.1	0.10		100.00
		0.10	0.12	0.12		100.00
			**Mean recovery % ± SD**			**100.42 ± 0.866**
MTP	99.12 ± 1.252	1.00	0.80	0.80		100.00
		1.00	1.00	1.00		100.00
		1.00	1.20	1.18		98.33
			**Mean recovery % ± SD**			**99.44 ± 0.632**

Logimax^®^ tablets (batch number 210361) are labeled to contain 5 mg FDP and 50 mg MTP

Bold represents the parameters and results that were further highlighted and focused on in the discussion of the results

^a^ Amount taken is 0.10 µg/mL of FDP and 1.00 µg/mL of MTP

^b^ Mean of five determinations of the dosage form assay

^c^ Mean of three determinations

### Evaluation of greenness of the developed HPLC-FD method

Method greenness is one of the crucial parameters that should be considered in developing new methods of analysis [40]. The optimum green assay is defined as the minimal usage of reagents and energy and the least waste formation. The green profile assessment of the developed method was done using the AGREE calculator, MoGAPI and RBG whiteness study then compared to the reported method [18].

The AGREE calculator is a simple and comprehensive tool for the evaluation of the green profile of analytical methods. The assessment depends on 12 principles of green analytical chemistry and has given a scale from 0 to 1. The results are represented as a pictogram with a circle in the middle that shows the final score. The intensity of the green color increases as the score gets close to 1.

**Fig. 4:**
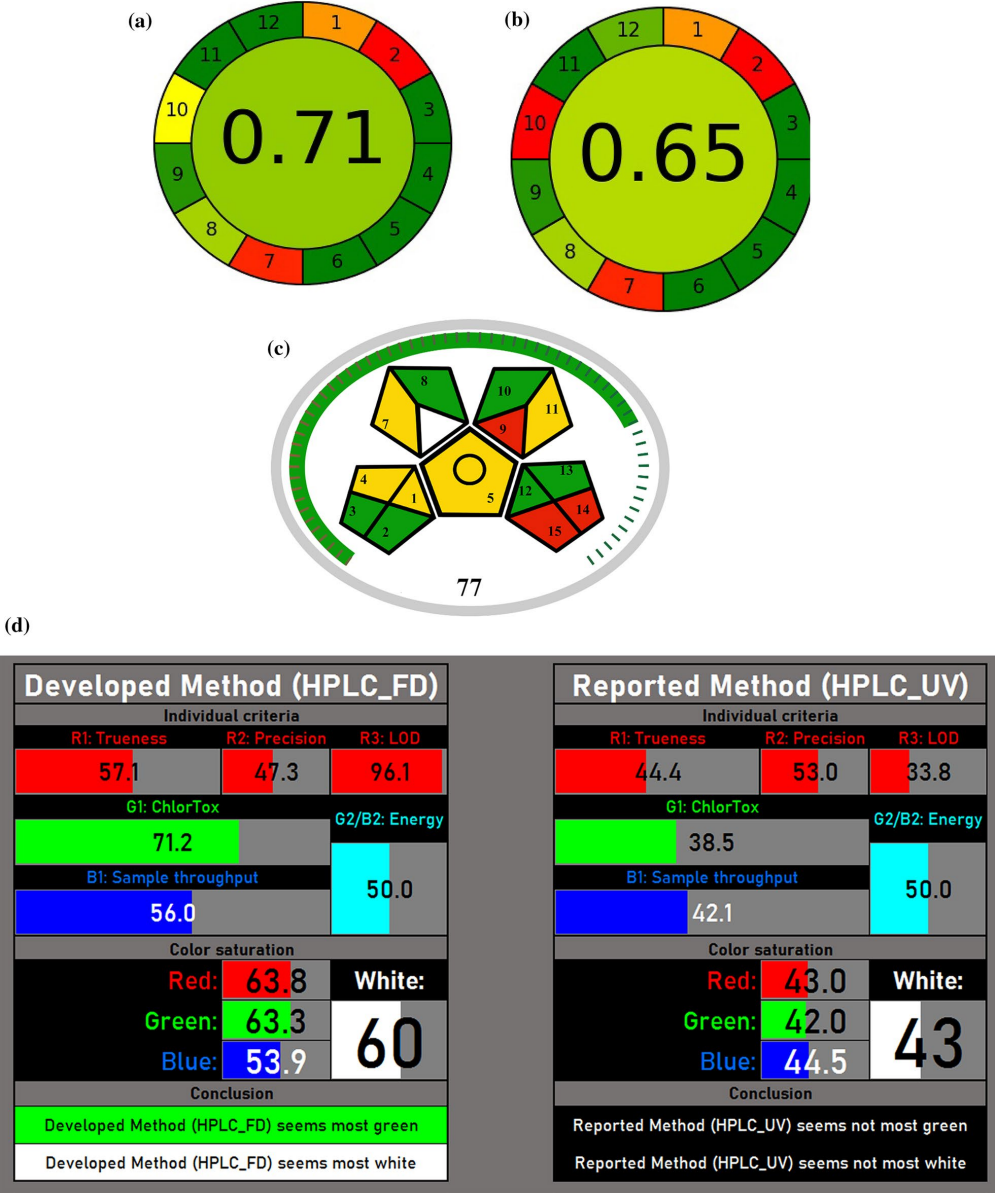

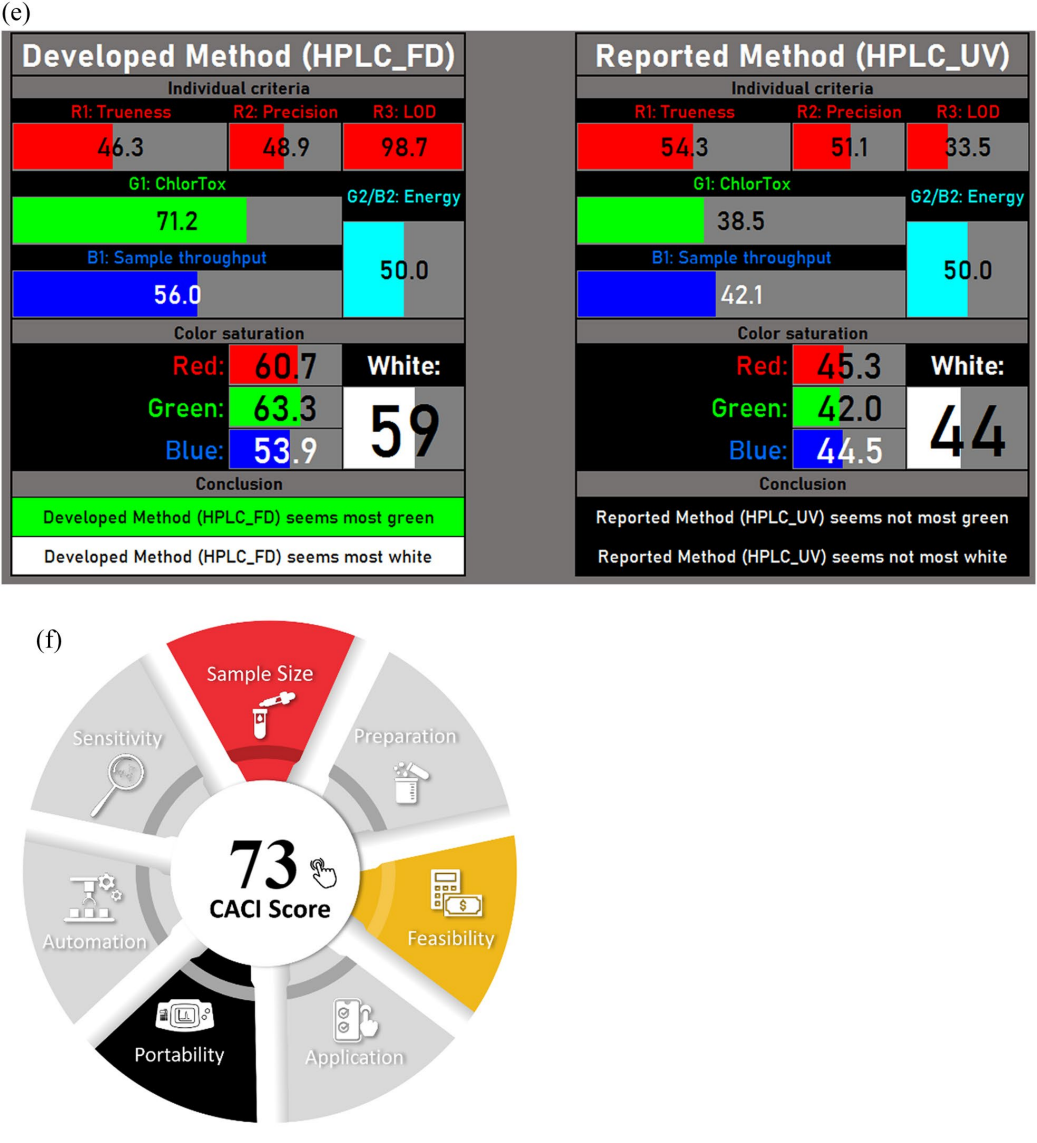
**a, b** AGREE calculator for the **a** proposed and **b** reported methods in biological matrix. **c** MoGAPI score for the proposed HPLC-FD method. **d** Automatically generated tables and pictograms were created using the RGBfast model to compare FDP in both methods. **e** Automatically generated tables and pictograms were created using the RGBfast model to compare MTP in both methods. **f** CACI score of the proposed HPLC-FD method

The pictograms obtained by the AGREE calculator for both the developed and reported method [18] in plasma is 0.71 and 0.65, respectively. As a result, the proposed method has displayed a higher green profile as shown in Fig. 4a, b.

Another method that was adopted was the MoGAPI that was retrieved from the analytical Procedure Index (GAPI) which is a tool designed to assess the environmental impact of analytical methods through five color-coded pentagrams, each representing different stages of the process: sampling, method type, sample preparation, solvent/reagent usage, and energy consumption. While GAPI provides a quick visual overview to evaluate and improve the greenness of methods, its main drawback is the lack of a total score, making it difficult to directly compare methods [41].

To address this limitation, the Modified GAPI (MoGAPI) tool and software were modified. MoGAPI combines the visual appeal of GAPI with a calculated total score, like the analytical Eco-Scale. Credits were assigned to each step based on its environmental impact, with higher scores given to greener practices of the method. The total score is then converted into a percentage, excluding non-applicable steps to ensure fairness. Greenness classification system categorizes methods as excellent green (≥ 75), moderately green (50–74), or insufficiently green (< 50) [41].

MoGAPI software was applied to evaluate the greenness approach of this proposed HPLC-FD method, providing a more precise and comprehensive assessment of its greenness profile impact. Evaluation of key aspects such as sampling, sample preparation, reagents and solvents, instrumentation, energy consumption, waste production and quantification were taken into consideration and incorporated into the software to generate a visual pentagram with a greenness score of 77% that signifies the method with high green profile shown in Fig. 4c.

An RGBfast method was applied to study and evaluate both the greenness and whiteness profile of the proposed HPLC-FD method and has been compared to the reported method [18]. RGBfast is a simplified evaluation tool to assess the environmental impact of analytical methods. It uses color codes, red for less green, green for eco-friendly, and blue for balanced profiles to rate methods. This simple approach aids researchers in assessing and improving sustainability. The RGBfast model utilizes an automated Excel spreadsheet where analysts can input relevant data. It includes guidelines for assessment and displays the results through automatically formatted tables and pictograms. The RGBfast model evaluates analytical methods using six criteria. Three red criteria focus on validation: trueness (R1) recovery as relative error, precision (R2) as relative standard deviation, and limit of detection (R3) as the LOD. For greenness (G1), the ChlorTox Scale measures chemical risk based on the amount and hazards of reagents, with calculations done in an Excel tool. Energy use (G2/B2) is assessed for both carbon footprint and cost, while sample throughput (B1) estimates the number of analyses that can be carried out in 24 h. This simplified model balances effectiveness, sustainability, and efficiency, making evaluations quicker and easier [42]. The excel spreadsheet was performed to the proposed HPLC-FD method and the reported method [18] to compare each of FDP and MTP in both methods, results are displayed in Fig. 4d, e. The developed method displayed a higher green and white profile than the reported one.

The HPLC-FD method for FDP and MTP was evaluated using three tools AGREE, MoGAPI, and RGBfast and all confirmed its green and sustainable nature. AGREE scored the method highly for its low solvent use and reduced environmental impact. MoGAPI rated the method positively for its energy efficiency and environmentally friendly design. RGBfast highlighted its low hazards, significant environmental advantages, and strong analytical performance. Overall, these tools demonstrate that the method is eco-friendly.

### Practicality and applicability of the proposed HPLC-FD method

This study introduces the Click Analytical Chemistry Index (CACI), a user-friendly metric and a software tool that was designed for evaluating analytical methods, emphasizing practicality and usability. It evaluates crucial key factors presented in Table S1 to determine how effectively a method meets these standards. Before CACI application, method validation is essential. The performance of the method is presented through a color-coded pictogram: colored sections indicate excellent performance, gray reflects moderate performance, and black represents poor performance or non-compliance. This approach allows analysts to identify a method's strengths and weaknesses [43]. CACI score of the proposed HPLC-FD method was found to be 73 that is acceptable in terms of practicality shown in Fig. 4f.

### Comparison with reported methods

Statistical studies were carried out to compare the results obtained by the proposed method to determine FDP and MTP simultaneously in their pure forms with the reported HPLC method [18]. The calculated *t* and *F* values were lower than the tabulated ones at a confidence limit of 95%, indicating no significant difference between both methods regarding accuracy shown in Table 9.

**Table 9:** Statistical comparison between the results acquired by the proposed HPLC-FD method and the reported HPLC method [18] of FDP and MTP

Parameter	FDP		MTP	
	Proposed	Reported**	Proposed	Reported**
Mean	99.31	98.85	99.38	100.45
SD	0.803	0.792	0.852	0.942
Variance	0.645	0.627	0.726	0.888
F test (6.39)*	1.029		1.223	
Student’s t test(2.306)*	0.926		1.882	

*Values between parenthesis represent the theoretical values of *t* and *F* tests at level of significance *P* = 0.05 and n = 5

** HPLC method composed of mobile phase of Methanol: Water: Acetonitrile (70:22:8, by volume) at a flow rate of 0.9 mL/min and UV detection at 260.0 nm for both drugs

## Conclusion

The proposed HPLC-FD method for the simultaneous estimation of FDP and MTP, along with tramadol (TDL) as the internal standard, is a novel bioanalytical approach. It has been demonstrated to be accurate, precise, sensitive, and selective**,** with a high green profile, making it suitable for analyzing the target drugs in pure form, pharmaceutical preparations, and biological matrices**.**

Compared to previously reported methods, the proposed method offers significant advantages. The previously reported method [21] did not include bioanalytical validation on spiked biological matrices following FDA guidelines, whereas our method complies with these standards. Additionally, while LC-MS/MS has been applied to drug analysis in dog plasma, it is costly and complex, whereas the proposed HPLC-FD method is simpler, more cost-effective, and highly sensitive. Furthermore, a comprehensive stability study was performed in our work, unlike the previously reported LC-MS/MS method [23].

The application of this method for bioanalytical validation in spiked human plasma has confirmed its suitability for bioequivalence and pharmacokinetic studies. It provides high sensitivity, acceptable recoveries, and low %RSD values, making it a promising tool for therapeutic drug monitoring. Although early elution of MTP was observed, the method still ensured acceptable recoveries in pure form, pharmaceutical dosage forms, and spiked human plasma. Several optimization attempts including the use of cationic and anionic ion-pairing agents, pH adjustments, and flow rate modifications were explored to delay MTP elution, but these did not significantly improve retention times. Nonetheless, the method remains highly effective for the intended analytical applications.

## Supplementary Information

Additional file 1.

## Data Availability

The data that has been analyzed and used in this study has been presented in this research and any other data will be available from the corresponding author upon reasonable request.
