# Supplementary material for: An eco-friendly bioanalytical RP-HPLC method coupled with fluorescence detection for simultaneous estimation of felodipine and metoprolol
Source: BMC Chem. 2025 May 23;19(1):141. doi: 10.1186/s13065-025-01507-0 (PMC12102954; doi:10.1186/s13065-025-01507-0)
Supplement: Supplementary file 1 — Additional file 1. [file 13065_2025_1507_MOESM1_ESM.docx]

**Supplementary data**

**Figure S1**: Effect of different pH in the mobile phase on retention time of FDP and MTP.

**Figure S2**: Effect of Ethanol% in the mobile phase on retention time of FDP and MTP.


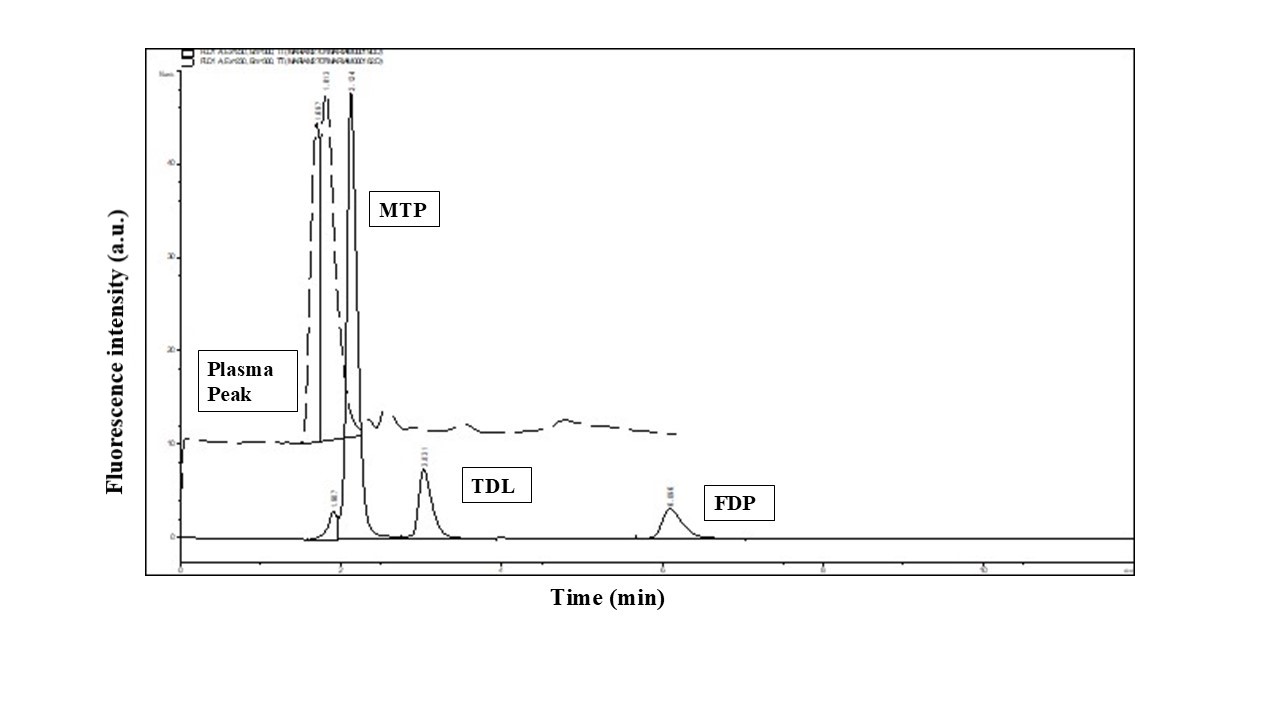


**Figure S3:** Overlay of chromatograms showing blank plasma chromatogram (---) and spiked plasma ( ) with MTP (0.50 µg/mL), tadalafil, TDL (0.20 µg/mL), and felodipine, TDF (0.50 µg/mL).

(S4,a)

(S4,b)

**Figure S4 (a,b)**: Calibration curves of (a) FDP and (b) MTP in their pure forms.

**Table S1:** Points system of CAC for proposed HPLC-FD method displaying the characteristics of the method

| **Method Characteristics** | |
| --- | --- |
| **Sample size** |  |
| What is the required sample size (in ml or g)? | **<1** |
| **Sample Preparation** |  |
| What level of preparation is required? | **Minimal sample preparation** |
| How much time is needed for sample preparation (in minutes) | **20** |
| **Feasibility** |  |
| Are the chemicals and reagents readily available? | **Commercially** |
| Are the required instruments available in laboratories? | **All equipment available in regular labs** |
| What is the total cost of analysis per sample? | **<10$ per sample** |
| **Application** |  |
| What is the application type of the method? | **Quantitative** |
| How many analytes can be tested? | **2-3 analytes** |
| How many matrices can be tested? | **2-3 matrices** |
| **Portability** |  |
| How portable is the method’s instrumentation? | **Not portable nor miniaturized** |
| **Automation** |  |
| What is the level of automation of this method? | **Semi-automatic** |
| **Sensitivity** |  |
| What is the method’s sensitivity level? | **≤ 10% of target concentration** |
| **Sample analysis time** |  |
| How long does the sample analysis take (in minutes)? | **6** |
